# Evidence for the Need to Evaluate More Than One Source of Extracellular Vesicles, Rather Than Single or Pooled Samples Only, When Comparing Extracellular Vesicles Separation Methods

**DOI:** 10.3390/cancers13164021

**Published:** 2021-08-10

**Authors:** Sarai Martinez-Pacheco, Lorraine O’Driscoll

**Affiliations:** 1 School of Pharmacy and Pharmaceutical Sciences, Panoz Institute, Trinity College Dublin, Dublin, Ireland; 2 Trinity Biomedical Sciences Institute, Trinity College Dublin, Dublin, Ireland; 3 Trinity St. James’s Cancer Institute, Trinity College Dublin, Dublin, Ireland

**Keywords:** extracellular vesicles, separation, enrichment, comparison of methodologies, characterization

## Abstract

**Simple Summary:**

Extracellular vesicles (EVs) are packages of information released from cells and are often described as mini maps of their cells of origin. EVs have many uses. For example, those found in the blood of cancer patients may inform us about tumor growth, spread, immune suppression, and drug response/resistance. EVs from other sources, inducing certain types of cells grown under laboratory conditions, have potential as therapeutics and drug delivery vehicles. For this reason, a few studies have compared methods of collecting EVs, but typically using only one EV source for the comparison. We had concerns that testing one source and extrapolating to others may not be adequate. To test this, we selected three HER2-positive breast cancer cell lines that grow in exactly the same type of fluid. We collected the fluid and tested two ways of separating the EVs from the fluid. We studied them based on seven characteristics. Both EV separation methods provided reproducible results for any of the given cell lines. However, the characteristics of the EV isolates were cell line- and method-dependent. Thus, we recommend not relying on a single EV source when comparing and selecting separation techniques for fundamental research or exploitation for clinical utility.

**Abstract:**

To study and exploit extracellular vesicles (EVs) for clinical benefit as biomarkers, therapeutics, or drug delivery vehicles in diseases such as cancer, typically we need to separate them from the biofluid into which they have been released by their cells of origin. For cultured cells, this fluid is conditioned medium (CM). Previous studies comparing EV separation approaches have typically focused on CM from one cell line or pooled samples of other biofluids. We hypothesize that this is inadequate and that extrapolating from a single source of EVs may not be informative. Thus, in our study of methods not previous compared (i.e., the original differential ultracentrifugation (dUC) method and a PEG followed by ultracentrifugation (PEG + UC) method), we analyzed CM from three different HER2-positive breast cancer cell lines (SKBR3, EFM192A, HCC1954) that grow in the same culture medium type. CM from each was collected and equally divided between both protocols. The resulting isolates were compared on seven characteristics/parameters including particle size, concentration, structure/morphology, protein content, purity, detection of five EV markers, and presence of HER2. Both dUC and PEG + UC generated reproducible data for any given breast cancer cell lines’ CM. However, the seven characteristics of the EV isolates were cell line- and method-dependent. This suggests the need to include more than one EV source, rather than a single or pooled sample, when selecting an EV separation method to be advanced for either research or clinical purposes.

## 1. Introduction

Extracellular vesicles (EVs) are lipid-bilayer-enclosed nanoparticles released by most, if not all, cells. The EVs’ cargo may include proteins, RNAs, DNA, and lipids, and they are often described as mini-maps of their cells of origin. The EVs’ bioactive cargo is instrumental in their role in cell-to-cell communication, mediating a broad range of physiological and pathological activities [1,2,3,4,5]. EVs have traditionally been categorized based on size and sub-cellular origin [6], with those derived from multi-vesicular bodies and having a size of approximately 30–150 nm termed exosomes, while those originating by budding/pinching from the cell membrane and typically have a size greater than 150 nm considered to be microvesicles [6,7]. EVs are detectable in a broad range of biofluids including cultured cells’ conditioned medium (CM), blood plasma and serum, milk, urine, saliva, and cerebrospinal fluid [8,9]. These important sources of EVs are commonly studied, but we must accept that once in a biofluid, we cannot claim the EVs’ exact origin and exit route(s) from their donor cells. Thus, the generally accepted collective term for exosomes and microvesicles is EVs.

Substantial effort has been invested by the EV community in establishing guidelines on minimal information for studies of extracellular vesicles (MISEV2018) and on supporting transparent reporting and centralizing knowledge in EV research to achieve increasing rigor and reproducibility of the knowledge generated [10,11]. It is generally accepted that, in many studies, EVs are not always completely isolated as pure EVs from other materials that exist in their biofluid and so the preferred term by many is “EV separation” or “EV enrichment”, rather than isolation; thus, these are the terms we will use here. Furthermore, as one EV separation method does not fit all purposes, understandably, there is no consensus on the optimal EV separation method. Interestingly, however, a survey performed in 2019 showed that although ultracentrifugation (UC)-based methods derived from the protocol described by Théry et al. [12] remain the most popular EV separation methods [13], the comparison between the 2019 survey and a survey performed in 2016 showed a significant (*p* < 0.05) increase in the use of precipitation methods [13,14]. Many such precipitation methods are claimed to represent user-friendly approaches, but the substantial trade-offs may be the reduced purity of isolates (as most published studies of precipitation methods use commercial kits that simply precipitate almost all content from the biofluid of interest), the substantial costs of kits, and the lack of information on the exact make-up of the kits’ precipitant, which would prevent the progress to utility that involves regulatory bodies. Bridging these issues, at least in part, a polyethylene glycol (PEG)-based method was developed that combines PEG-based precipitation and UC, resulting in EV enriched samples that are suitable for downstream functional in vivo pre-clinical studies [15]. Thus, we were interested in directly comparing the more traditional differential UC (dUC) method with the PEG followed by UC (PEG + UC) method.

**Figure 1:**
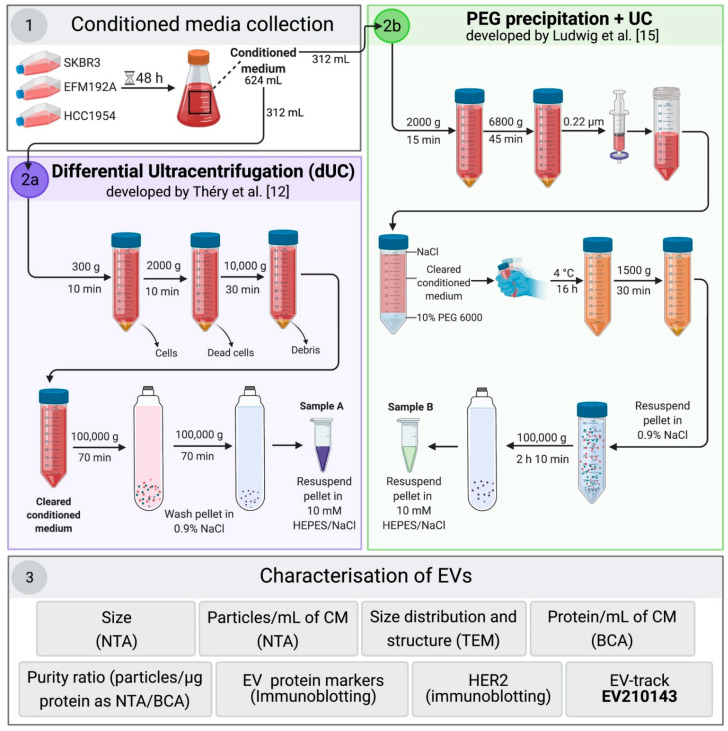
Flow diagram of methodology used. (**1**) SKBR3, EFM192A, and HCC1954 cells were seeded in complete medium and allowed to attached overnight. Complete medium was then replaced with medium containing EV-depleted FBS (dFBS). After 48 h incubation, conditioned medium (CM) was collected and cells were counted. Each CM batch was divided into two equal volumes and used for the EV-enrichment method comparison (i.e., (**2a**) 312 mL of CM was used for EV separation by differential ultracentrifugation (dUC); (**2b**) the other 312 mL were used for PEG-based method followed by ultracentrifugation (PEG + UC)). The diagram shows the step-by-step of each approach used for EV separation/enrichment and (**3**) the parameters evaluated during the subsequent characterization of the isolates. The process was conducted a total of *n* = 3 times. Schematic representation was created with BioRender.com (accessed on 6 August 2021).

Although the EV separation methods compared here have not previously been compared, many good publications have arisen from studies comparing other EV separation techniques. However, typically, these comparison studies, whether they include CM [16,17] or other biofluids such as blood [18,19], involve using one pooled source of EVs. For our study, comparing PEG + UC to the traditional dUC method, we hypothesized that simply analyzing one pooled sample may not be adequate for a fully informed comparison and that a range of similar, but different, sources of EVs should be included. Thus, to test our hypothesis, we compared EV isolates from CM of three different HER2-positive breast cancer cell lines that grow in the same medium type. A graphical representation of both EV separation methods and the subsequent characterization methods used is summarized in Figure 1.

## 2. Materials and Methods

We submitted all relevant data of our experiments to the EV-TRACK knowledge base (EV-TRACK ID: EV210143) [11].

### 2.1. Cell Culture

Three HER2-positive breast cancer cell lines (SKBR3, EFM192A and HCC1954) were routinely maintained in RPMI-1640 medium (Sigma-Aldrich, Burlington, MA, USA; Cat. #: R0883) supplemented with 10% fetal bovine serum (FBS) (Thermo Fisher Scientific, Waltham, MA USA; Cat. #: 10270-106), and 2 mM L-Glutamine (Sigma-Aldrich, Cat. #: G7513) as complete medium. All cells were cultured at 37 °C with 5% CO_2_ and routinely tested to ensure that they were free of *Mycoplasma* contamination. 

### 2.2. EV Preparation from Conditioned Cell Medium

Before conditioned medium (CM) collection, 26 × T175 cm^2^ flasks of SKBR3, EFM192A and HCC1954 cells, respectively, were seeded in complete RPMI-1640 medium. The medium was replaced the next day with RPMI-1640 medium containing 10% EV-depleted-FBS (dFBS), 2 mM L-Glutamine, and 1% penicillin/streptomycin (Sigma-Aldrich, Cat. #: P4333). After the cells conditioned the medium for 48 h, approximately 624 mL aliquots of CM were collected from each cell line and divided into equal volumes to progress through the two protocols illustrated in Figure 1. Cells were counted and their viability checked, showing all cultures to be ≥95% viable.

### 2.3. EV Isolation by Differential Ultracentrifugation

For the dUC approach, separation of EVs was performed following the protocol described by Théry et al. [12]. Briefly, 312 mL freshly harvested CM was spun at 300× *g* for 10 min (5810R centrifuge, Eppendorf, Hamburg, Germany). The resulting supernatant was then centrifuged at 2000× *g* for 10 min. After any dead cells were removed, the CM was further spun at 10,000× *g* for 30 min at 4 °C in a 5810R centrifuge. The supernatant was then transferred to 8 × 39 mL Quick-Seal^®^ polypropylene centrifuge tubes (Beckman Coulter, Brea, CA, USA; Cat. #: 342414) and spun at 100,000× *g* for 70 min at 4 °C in an Optima XPN-100 Ultracentrifuge using a Type 70 Ti fixed-angle rotor (Beckman Coulter). The resulting eight pellets with EVs were washed in a combined total of 39 mL of 0.9% NaCl (Sigma-Aldrich, Cat. #: 71376) and ultracentrifuged exactly as previously performed. The final EV pellet was resuspended in 100 μL of 10 mM HEPES/NaCl (Thermo Fisher Scientific, Cat. #:15630049) and stored at −80 °C.

### 2.4. EV Precipitation by PEG + UC Washing Step

For the PEG + dUC approach, separation of EVs was performed following the protocol described by Ludwig et al. [15]. Briefly, 312 mL CM were spun at 2000× *g* for 15 min and then 6800× *g* for 45 min (5810R centrifuge, Eppendorf) and supernatants were filtered through a 0.22-μm membrane filter (Thermo Fisher Scientific, Waltham, MA, USA; Cat. #:15206869). A stock solution of PEG 6000 (50% (*w/v*)) (Sigma-Aldrich; Cat. #: 81260) was added to the supernatants for a final concentration of 10% PEG and 75 mM NaCl (Sigma-Aldrich, Cat. #: 71376). After inverting the tubes thrice to allow the samples to mix, the samples were stored at 4 °C for 16 h. Post 16 h incubation, samples were spun at 1500× *g* for 30 min at 4 °C (5810R centrifuge, Eppendorf), and the supernatants were removed. To remove PEG residues, the pellets were combined and washed with 39 mL of 0.9% NaCl and transferred in 39 mL Quick-Seal^®^ polypropylene centrifuge tubes (Beckman Coulter, Cat. #: 342414). Ultracentrifugation was performed using a Type 70 Ti fixed-angle rotor in an Optima XPN-100 Ultracentrifuge (Beckman Coulter) at 110,000× *g* for 2 h 10 min at 4 °C. The final EV pellet was resuspended in 100 μL of 10 mM HEPES/NaCl (Thermo Fisher Scientific, Cat. #:15630049) and stored at −80 °C.

### 2.5. Nanoparticle Tracking Analysis (NTA)

Average size distribution and particle concentration analyses of the EV-enriched isolates were performed using a NanoSight NS500 system (Malvern Instruments Ltd., Malvern, UK). Brownian motion of the particles was captured at 30 frames/s speed. For this, EV samples were diluted using filtered Dulbecco’s Phosphate Buffered Saline (DPBS) (Sigma-Aldrich, Cat. #D8537), loaded onto the NTA using a NanoSight syringe pump, and videos of the particles were recorded and analyzed using NTA version 3.3 software. Aliquots of the same filtered PBS were used as the control.

### 2.6. Protein Content

The protein content of the EV samples was determined using the Micro BCA™ Protein Assay Kit (Thermo Fisher Scientific, Cat. #23235). Protein analysis was performed according to the recommendations of the manufacturer using the 96-well plate procedure.

### 2.7. Immunoblotting

Cells and EV isolates were lysed using Cell Lysis Buffer (Thermo Fisher Scientific, Cat. #: FNN0011) supplemented with protease inhibitor cocktail (Roche, Basel, Switzerland; Cat. #: 04693116001). Ten μg of cell lysates and EV lysates were loaded onto 10% Mini-PROTEAN TGXTM gels (Bio-Rad Laboratories, Hercules, CA, USA; Cat. #: 4561034) and the protein was transferred to PVDF membranes (Bio-Rad Laboratories; Cat. #: 1620177). Following the transfer, the membranes were blocked with 5% (*w/v*) BSA in PBS containing 0.1% Tween-20 (PBS-T) and incubated overnight at 4 °C with primary anti-human antibodies to HER2 (1:1000; Calbiochem, San Diego, CA, USA; Cat. #: OP15), CD63 (1:500; Abcam, Cambridge, UK; Cat. #: ab68418), syntenin (1:1000; Abcam, Cat. #: ab133267), calnexin (1:1000; Abcam, Cat. #: ab133615), CD9 (1:1000; Abcam, Cat. #: ab92726), or GRP94 (1:1000; Cell Signaling Technology, Danvers, MA, USA; Cat. #: 2104S). After washing thrice with PBS-T, the membrane was incubated with anti-rabbit (1:1000; Cell Signaling Technology, Cat. #: 7074) or anti-mouse (1:1000, Cell Signaling; Cat. #: 7076) secondary antibodies in 5% BSA/PBS-T for 1 h at RT and imaging was performed using an automated Chemidoc exposure system (Bio-Rad Laboratories) and using the SuperSignal West Femto Chemiluminescent Substrate Kit (Thermo Fisher Scientific, Cat. # 11859290) or SuperSignal West Femto Maximum Sensitivity Substrate (Thermo Fisher Scientific, Cat. #: 34096) for detection. Cell lysate (CL) from the individual cell line of origin was included in all gels as the control and densitometric analysis was performed using Fiji software.

### 2.8. Transmission Electron Microscopy

Samples were prepared from transmission electron microscopy (TEM) analysis following our published protocol [20,21] that was adapted from a previous publication [22]. Briefly, 10 μL of the sample was placed onto carbon-coated grids (Ted-Pella B 300 M, Mason Technology Ltd., Dublin, Ireland; Cat. #: 01813-F) and incubated for 10 min at RT. After incubation, samples were fixed with 4% glutaraldehyde and contrasted with 2% phosphotungstic acid. The grids were examined at 100 kV using a JEOL JEM-2100 TEM (JOEL USA Inc., Peabody, MA, USA).

### 2.9. Statistical Analysis

All results presented were obtained from three independent experiments, starting each time with seeding a new batch of cells. Paired t-test analysis was performed using GraphPad Prism version 9.1.9 for macOS (GraphPad Software Inc., La Jolla, CA, USA). Data are expressed as means ± standard error of the mean (SEM). * *p* < 0.05; ** *p* < 0.01; *** *p* < 0.001.

## 3. Results

### 3.1. Particle Size and Yield of EVs Separated from CM Differ Based on the Separated Method and CM Source

**Figure 2:**
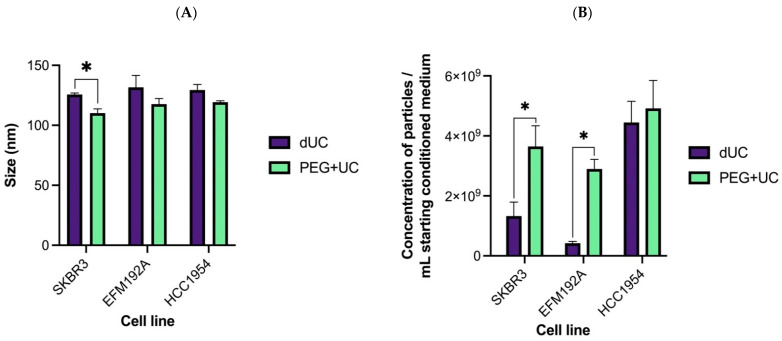
Characterizing particles/EVs, released by HER2-positive cell lines and harvested using differential ultracentrifugation (dUC) or polyethylene glycol-based precipitation followed by ultracentrifugation (PEG + UC), by NTA. (**A**) Particle size mode estimations. (**B**) Quantification of particle/EV numbers and normalizing to mL of CM. Results represent n = 3 isolates ± SEM, * *p* < 0.05.

**Figure 3:**
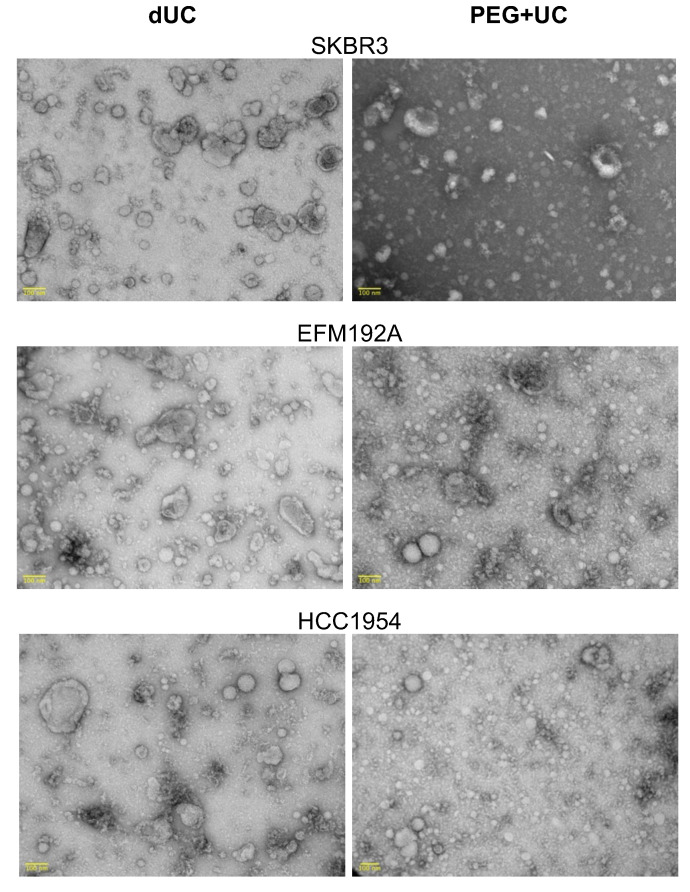
Characterizing particles/EVs released by HER2-positive cell lines and harvested using differential ultracentrifugation (dUC) or polyethylene glycol-based precipitation followed by ultracentrifugation (PEG + UC) by TEM. Scale bar = 100 nm.

The average sizes and particle concentration of the products separated from CM by each method, dUC and PEG + UC, and analyzed by NTA are shown in Figure 2. Isolates obtained from SKBR3 CM using the PEG + UC approach were significantly smaller than those obtained using the dUC method (Figure 2A). No significant differences in sizes were found for the HCC1954 and EFM192A isolates when comparing the two methods. It is noteworthy, however, that the qualitative TEM approach showed that both methods produced a range of particle sizes from CM of each of the three cell lines (Figure 3). The NTA representative size distribution graphs with the mean and modal sizes are provided in the Supplementary Materials (Figure S1), with the average sizes and particle concentration of the samples detailed in Table S1.

Regarding the particle yield as determined by NTA, with CM from two of the three cell lines (i.e., EFM192A and SKBR3), significantly higher particle concentrations were obtained by the PEG + UC compared to the dUC method (Figure 2B). Specifically, the largest and most significant difference was found with the EFM192A samples (PEG + UC versus dUC: 6.9-fold; *p* = 0.0145), followed by the SKBR3 samples (2.75-fold; *p* = 0.0234). With the HCC1954 CM isolates, there was no significant difference whether they resulted from dUC or PEG + UC.

### 3.2. Protein Quantification Present after EV Separation from Conditioned Medium and Purity

**Figure 4:**
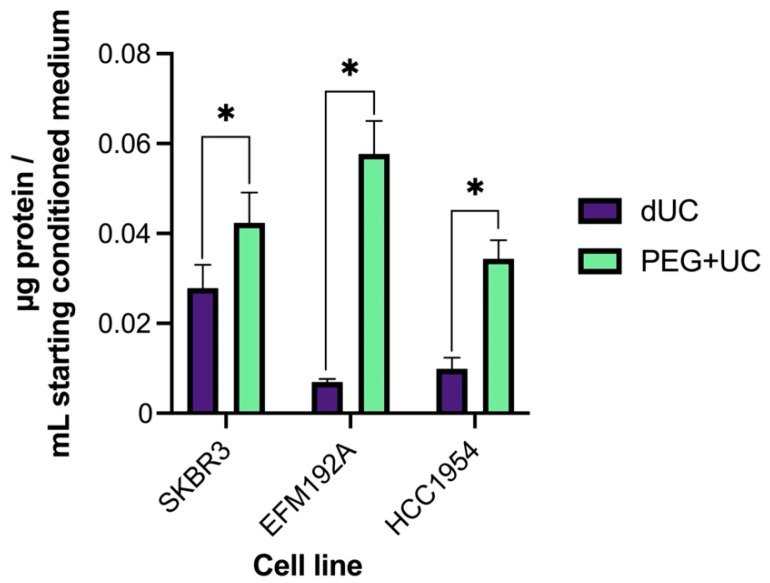
Protein quantification of isolates. Mean values of protein concentrations (µg of protein/mL of starting conditioned medium) of n = 3 isolates ± SEM are illustrated, * *p* < 0.05.

As protein quantitates are sometimes analyzed as a surrogate for EVs, here the relative amounts of protein (expressed as µg of protein/mL starting CM) present in each isolate was determined by the Micro BCA™ Protein Assay Kit (Figure 4). These were found to differ significantly between the isolates of all three cell lines, always being highest in the samples obtained via the PEG + UC method.

**Figure 5:**
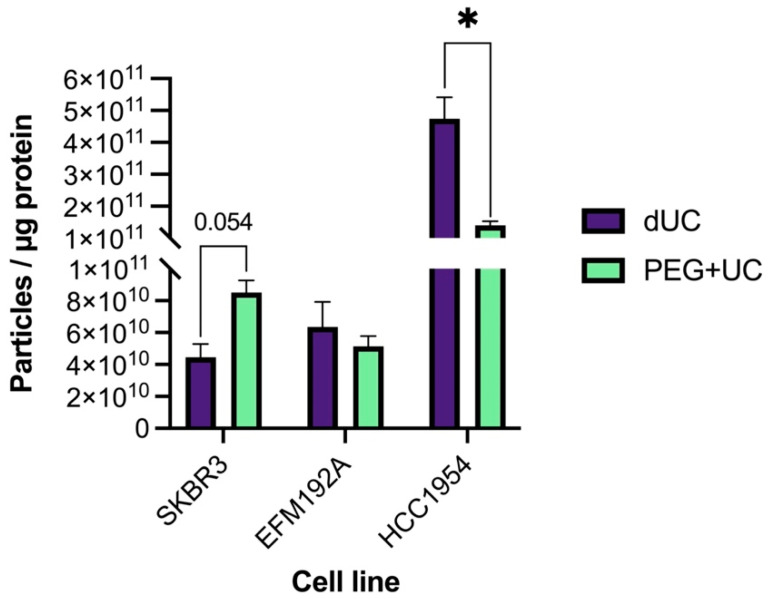
Particle to protein ratio to estimate EV purity. Mean values of particles to protein ratios (particles/µg of protein) of *n* = 3 isolates ± SEM are illustrated, * *p* < 0.05.

The ratio of the particle counts to protein concentration (particle/protein ratio; P/μg) obtained using tools such as NTA and BCA, respectively, have been proposed as straightforward methods to estimate the purity of EVs [23]. That study established that ratios greater than 3 × 10^10^ P/μg are associated with high vesicular purity; ratios between 2 × 10^10^–2 × 10^9^ P/μg are considered low purity; and ratios <1.5 × 10^9^ P/μg are unpure. As illustrated in Figure 5, all the samples analyzed in this study—regardless of whether separated by dUC or PEG + UC—showed a ratio higher than 3 × 10^10^ P/μg and so would be considered to be of high purity. However, a relatively greater purity was achieved with dUC (4.74 × 10^11^ ± 6.73 × 10^10^) compared to PEG + UC (1.41 × 10^11^ ± 1.21 × 10^10^) for the HCC1954 samples. Conversely, the opposite was found with the SKBR3 samples (4.45 × 10^10^ ± 8.40 × 10^9^ and 8.52 × 10^10^ ± 7.61 × 10^9^, respectively), where a higher purity ratio resulted in the samples obtained by the PEG + UC compared to the dUC approach, although statistical significance was not reached (*p* = 0.054). 

### 3.3. Presence of EV Specific Markers and HER2 Varied Depending on the Cells of Origin and EV Separation Method Applied

In keeping with MISEV2018 guidelines [10], immunoblotting analysis was performed on EV lysates and cellular lysates for the EV positive markers, CD63, syntenin, and CD9. Two proteins not considered to typically be enriched in EVs (i.e., GRP94 and calnexin) were also analyzed.

**Figure 6:**
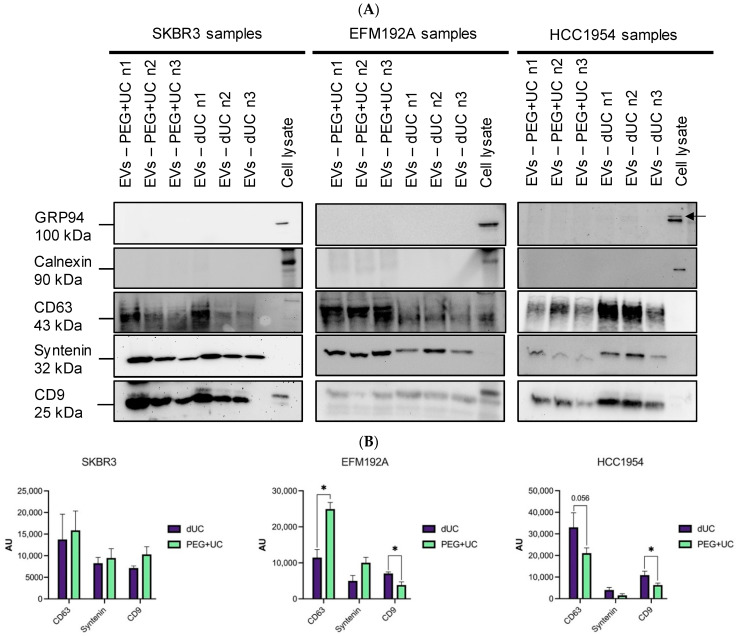
Immunoblots of EVs -positive and -negative markers present on the samples. (**A**) Ten µg of protein lysates (EVs from PEG + UC or dUC, or corresponding donor cell line) was loaded per lane and analyzed for GRP94, calnexin, CD63, syntenin, and CD9. Each blot represents three independent experiments and the densitometric analysis of these three are presented in (**B**) as the mean of n = 3 ± SEM, * *p* < 0.05.

Regardless of the EV separation method used, GRP94 and calnexin were not detected with any of the EV samples analyzed (Figure 6A). Both the dUC and PEG + UC generated samples were positive for all three EV positive markers (Figure 6A, with densitometric analysis of n = 3 presented in Figure 6B). Although equal quantities of protein were loaded on the gels, the enrichment of EV markers in isolates obtained was cell line- and EV separation method-dependent. For SKBR3, there were no significant differences detected. CD63 was found to be enriched in PEG + UC versus dUC isolates obtained from EFM192A CM. However, its presence was lower in the HCC1954 samples using PEG + UC compared to dUC, *albeit* not significantly. With EFM192A and HCC1954, significantly more CD9 was detected following dUC compared to PEG + UC, while no significant difference was found with CD9 for SKBR3.

**Figure 7:**
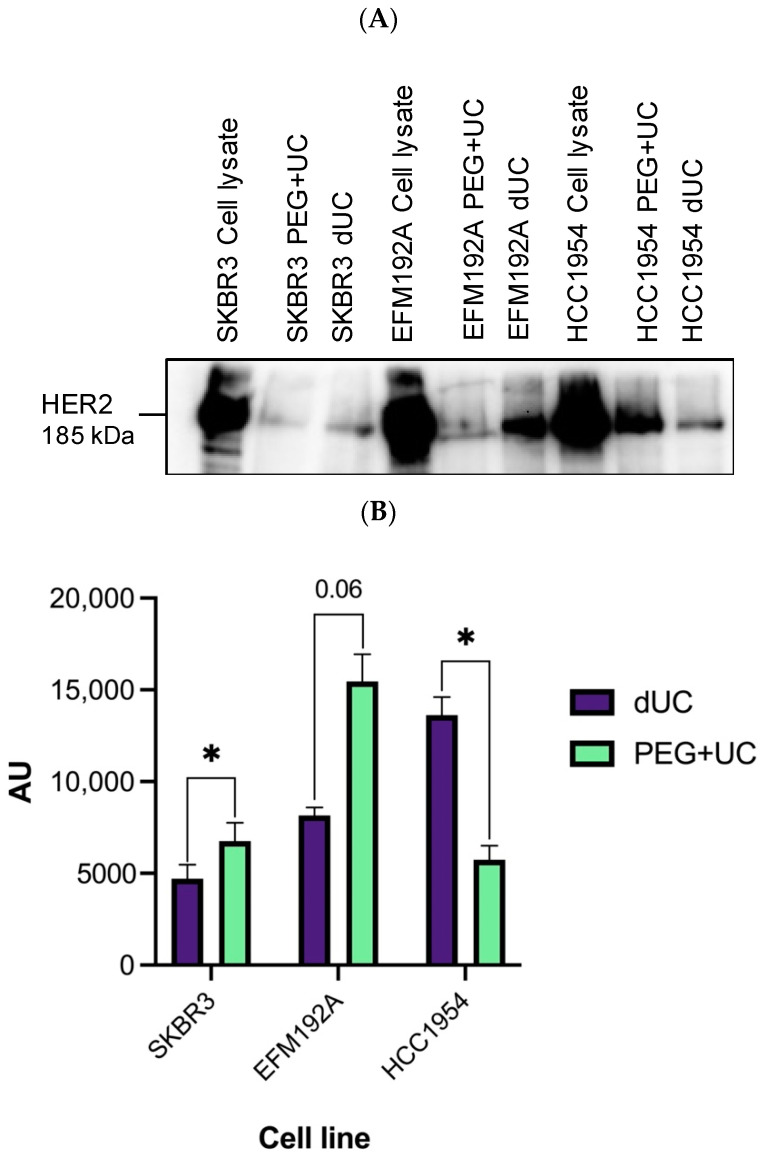
Immunoblots of HER2 markers present on the samples. (**A**) Ten µg of protein lysates (EVs from PEG + UC or dUC, or corresponding donor cell line) was loaded per lane and analyzed for HER2. This blot represents three independent experiments and the densitometric analysis of these three are presented in (**B**), with each bar representing the mean of the densities of the signals from *n* = 3 repeat experiments ± SEM, * *p* < 0.05.

As all three cell lines are HER2-positive, we investigated the presence of HER2 in these isolates (Figure 7). As for the EV markers, although equal quantities of protein were loaded on the gels, the enrichment of HER2 in the isolates obtained was cell line-/EV separation method-dependent. Specifically, for SKBR3, PEG + UC resulted in significantly more detectable HER2 compared to the isolates resulting from dUC. Conversely, for HCC1954, dUC compared to PEG + UC resulted in significantly more HER2.

Table 1 brings together the main observations from this study.

## 4. Discussion

EVs play an important role in normal and pathological mechanisms carrying functional molecules including proteins, lipids, and nucleic acids. Over recent years, interest in the EV field has increased considerably and EVs have been associated with numerous pathological processes as well as physiological roles including inflammation [24], tissue regeneration [25], osteogenesis [26], and hypoxia [27]. In cancer, for example, our group was the first to show that EVs can transmit resistance to anti-cancer drugs [28,29]. The potential of EVs, for example, from mesenchymal stem cells (MSCs), as therapeutics and natural drug delivery systems is also of substantial interest [4,25]. Areas in this field that need further attention in our effort toward exploiting the therapeutic potential of EVs, especially those released from cultured mammalian cells, include the EV separation/enrichment step [30,31].

As mentioned in the Introduction, indeed, a few EV separation comparison studies have been reported; *albeit* not comparing the specific methods included here. The consensus from those studies is that the method of EV separation used depends on the downstream application of the recovered EVs. While we are fully in agreement with this, we felt that it was important to take a further step back and establish whether information on the comparison of methods obtained from only one cell line’s CM can be assumed to be correct for others and so extrapolated. As we have summarized in Table 1, considering CM from three similar, but different, cell lines (i.e., three independent HER2-positive cell lines, cultured in the same type of medium), had we only worked with one of these—rather than all 3—and extrapolated, we would have produced some misleading results. It is important to note that this is not due to a lack of reproducibility when working with dUC or PEG + UC on any of the cell lines’ CM, as the results from all three independent repeat experiments were very reproducible for each of SKBR3, EFM192A, and HCC1954.

Specifically, in relation to the EV size recorded by NTA, PEG + UC resulted in smaller particles than those obtained by dUC for SKBR3, but not for EFM192A or HCC1954. In 2/3 cases (SKBR3 and EFM192A), PEG + UC resulted in a higher quantity of particles, but this was not so with HCC1954. One exception of the seven parameters considered (Table 1) was protein quantity, which possibly included protein “contaminants” and was significantly higher with PEG + UC vs. dUC. This was not unexpected, as the group who developed this protocol and applied it to CM from HEK293T cells [15] reported higher amounts of impurities/non-EV associated molecules when comparing their method to sucrose density gradients (GUC). Conversely, however, the gain with PEG + UC was that the isolates maintained therapeutic activity (unlike those derived by GUC) and those therapeutic activities did not seem to be as a consequence of the impurities. Having said that, in relation to impurities, if we consider the method reported by Webber and Clayton [23] to estimate impurities, the isolates obtained from all three cell lines’ CM using dUC or PEG + UC fell within the threshold of high vesicular purity, even if, within that range, the dUC isolates have a ratio value significantly higher than the PEG + UC isolates for one of the three cell lines (i.e., HCC1954). 

Regardless of the CM source or method employed, all EV samples were negative for GRP94 and calnexin, which are typically considered to be negative markers for EV; in keeping with the suggestion of—at most—minimal impurities being present. Conversely, all three proteins considered to be positive markers of EVs (i.e., CD63, syntenin, and CD9) were detected to different extents in all isolates, supporting the presence of EVs. The only significant difference observed following densitometry analysis of all blots with PEG + UC compared to dUC was a significant decrease in CD9 for both EFM192A and HCC1954 and a significant increase in CD63 for EFM192A. Regarding HER2, although all three cell lines are HER2-positive and HER2 was detected on all EV isolates (reflecting the status of their cancer cells of origin), the results obtained from the analysis of the products showed differences that were cell line-/method-dependent (i.e., comparing PEG + UC to dUC isolates). Specifically, for EFM192A, there was no significant difference in HER2 regardless of the method, while PEG + UC compared to dUC resulted in significantly more and significantly less HER2, respectively, for SKBR3 and HCC1954.

Altogether, our findings suggest that both dUC and PEG + UC have uses as reproducible methods for separating quite pure EVs, although PEG + UC tends to (but does not always) precipitate more particles and proteins that may not be of EV origin. However, importantly, what our study also shows is that consideration must be given to the inclusion of more than one source of CM (and so potentially the same for other biofluids) when EV separation techniques are being compared, rather than using a single or pooled sample that may not generate results that can be extrapolated to other samples.

## 5. Conclusions

Findings from the current study indicate that different EV sources should be included into comparison studies of EV separation methodologies in order to select the best methodology for a specific source of EVs.

## Figures and Tables

**Table 1:** Summary of the comparison of dUC and PEG + UC separation of EVs from the CM of three cell lines.

Characteristics/ Parameters Evaluated (Method)	Changes (Statistically Significant ↑ or ↓) with PEG + UC vs. dUC		
	SKBR3 Source	EFM192A Source	HCC1954 Source
Size (NTA)	↓ PEG + UC	NS	NS
Particles/mL of CM (NTA)	↑ PEG + UC	↑ PEG + UC	NS
Size distribution and structure (TEM)	Qualitative, so statistical analysis not possible	Qualitative, so statistical analysis not possible	Qualitative, so statistical analysis not possible
Protein/mL of CM (BCA)	↑ PEG + UC	↑ PEG + UC	↑ PEG + UC
Purity ratio (particles/μg protein as NTA/BCA)	NS	NS	↓ PEG + UC
EV protein markers (immunoblots)	CD63: NS Syntenin: NS CD9: NS GRP94: Undetected Calnexin: Undetected	CD63: ↑ PEG + UC Syntenin: NS CD9: ↓ PEG + UC GRP94: Undetected Calnexin: Undetected	CD63: NS Syntenin: NS CD9: ↓ PEG + UC GRP94: Undetected Calnexin: Undetected
HER2 (immunoblots)	↑ PEG + UC	NS	↓ PEG + UC

NS = not significant.

## Data Availability

Supporting data can be found in the Supplementary Materials.

## Supplementary Materials

The following are available online at https://www.mdpi.com/article/10.3390/cancers13164021/s1, Figure S1: Representative nanoparticle tracking analysis (NTA) images. Table S1: Sizes and particle yields; Table S2: Comparison of relative amount of protein on samples obtained by dUC vs. PEG + UC.
