# Supplementary material for: Evidence for the Need to Evaluate More Than One Source of Extracellular Vesicles, Rather Than Single or Pooled Samples Only, When Comparing Extracellular Vesicles Separation Methods
Source: Cancers (Basel). 2021 Aug 10;13(16):4021. doi: 10.3390/cancers13164021 (PMC8392213; doi:10.3390/cancers13164021)
Supplement: Supplementary file 1 [file cancers-13-04021-s001.zip › cancers-1320406-supplementary.pdf]

Supplementary Material

Evidence of the need to evaluate more than one source of extracellular vesicles, rather than single or pooled samples only, when comparing EV separation methods

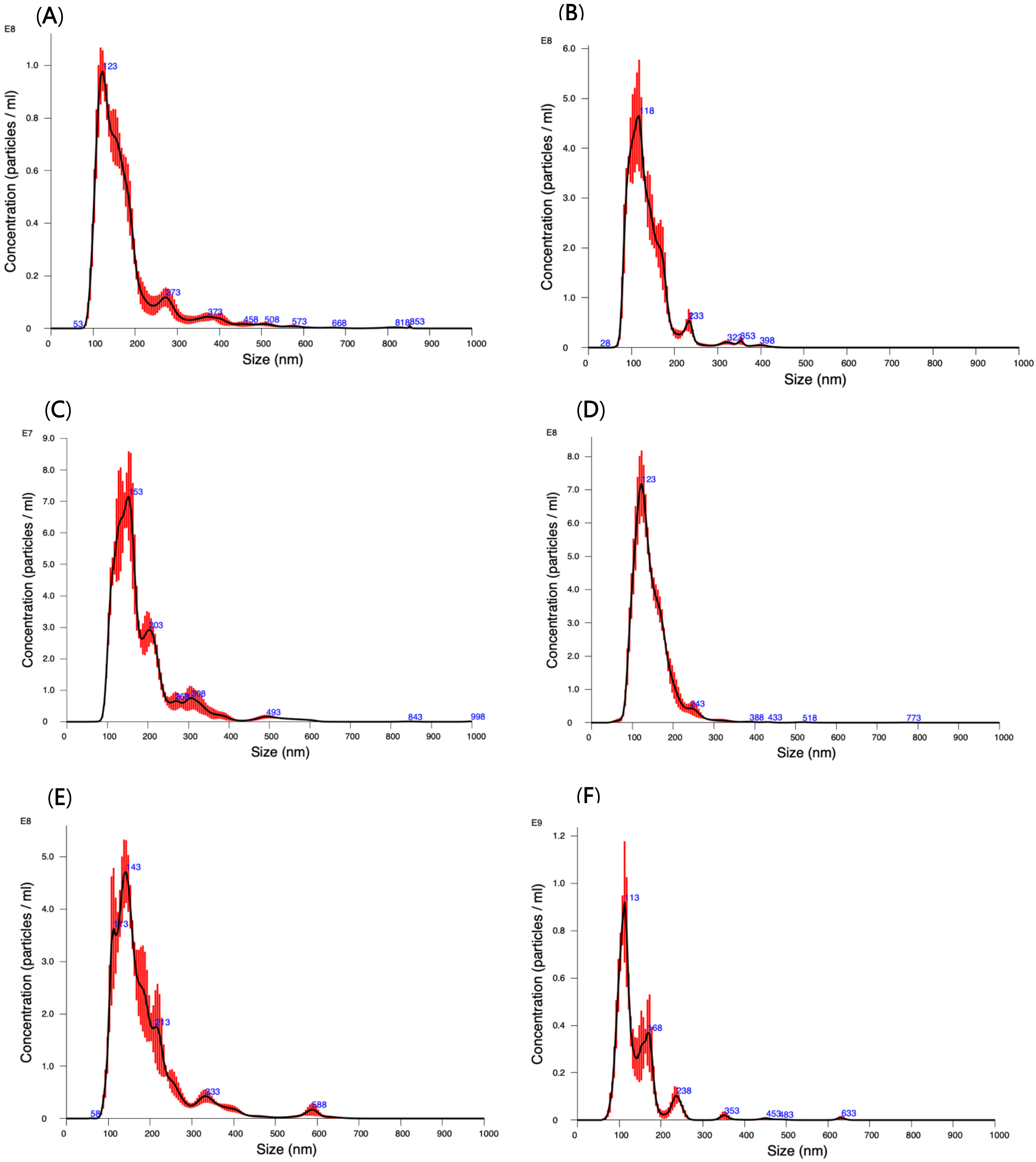

Figure S1. Representative nanoparticle tracking analysis (NTA) images. SKBR3 isolates obtained by (**A**) dUC and (**B**) PEG+UC; EFM192A isolates obtained by (**C**) dUC and (**D**) PEG+UC; HCC1954 isolates obtained by (**E**) dUC and (**F**) PEG+UC

Table S1. Sizes and particles yield, as analyzed by NTA

	Size (nm)			
	**dUC**	**PEG+UC**	**P value**	
**SKBR3**	125.7±2.0	110.2±6.0	0.0396*	
**EFM192A**	131.7±17.2	117.7±7.9	0.1214	
**HCC1954**	129.4±8.0	119.3±1.8	0.2105	
	**Particle concentration / mL starting conditioned medium**			
	**dUC**	**PEG+UC**	**Fold change**	**P value**
**SKBR3**	$1.33x10^9 \pm 4.66x10^8$	$3.65x10^9 \pm 6.9x10^8$	2.75	0.0234*
**EFM192A**	$4.20x10^8 \pm 6.37x10^7$	$2.99x10^9 \pm 3.20x10^8$	6.9	0.0145*
**HCC1954**	$4.45x10^9 \pm 7.08x10^8$	$4.92x10^9 \pm 9.30x10^8$	1.11	ns

Data are shown as mean ± SEM of n=3 and compared with paired t-test. *P<0.05

Table S2. Comparison of relative amount of protein normalized to mL of starting conditioned medium on samples obtained by dUC *versus* PEG+UC

	Relative amount of protein (μg of protein/mL starting conditioned medium)			
	**dUC**	**PEG+UC**	**Fold change**	**P value**
**SKBR3**	0.028 ± 0.005	0.042 ± 0.007	1.5	0.0131 (*)
**EFM192A**	0.007 ± 0.0007	0.06 ± 0.007	8.3	0.0167 (*)
**HCC1954**	0.010 ± 0.002	0.034 ± 0.004	3.5	0.0141 (*)

Data are shown as mean ± SEM of n=3 and compared with paired t-test. *P<0.05
